# Analysis of Extemporaneous Prescriptions Prescribed by Dermatovenerologists in Latvia and Comparison with Standardized Compounded Preparation Monographs of Germany and USA

**DOI:** 10.3390/medicina56010029

**Published:** 2020-01-10

**Authors:** Olga Kiseļova, Baiba Mauriņa, Venta Šidlovska

**Affiliations:** Department of Dosage Form Technology, Riga Stradins University, Dzirciema Street 16, LV-1007 Riga, Latvia; baiba.maurina@rsu.lv (B.M.); venta.sidlovska@rsu.lv (V.Š.)

**Keywords:** extemporaneous prescriptions, dermatovenerologists, compounding, Latvia

## Abstract

*Background and objectives*: Even though many industrially manufactured medicines are available, extemporaneous preparations still have their niche in dermatology. In several countries, dermatovenerologists are one of the specialists prescribing extemporaneous medicines the most. In order to increase the quality of compounded medications and minimize risks to patient safety, several countries, for example, Germany and the United States of America (USA), created standardized compounded preparation monographs. Latvia, unlike these countries, does not have any officially approved standardized compounded preparation monographs. The purpose of this survey is to analyze the extemporaneous prescriptions prescribed by Latvian dermatovenerologists to identify the active ingredients, combinations of active ingredients, and excipients prescribed by dermatovenerologists the most often, and to find out how many active ingredients are most often compounded in different dosage forms. To understand whether the extemporaneous formulations used in Latvia for dermatological indications are evidence-based, they were compared with German and USA formulations. *Materials and Methods*: A database was created entering data on all the prescriptions prepared in the selected pharmacies in 2017 to summarize information on extemporaneous prescriptions. The prescriptions prescribed by Latvian dermatovenerologists were selected and compared with German and USA formulations. *Results*: Data from 17 Latvian pharmacies were collected, and 2521 extemporaneous formulations were analyzed. In preparation of semi-solid dosage forms, 25 bulk drug substances and 37 industrially manufactured preparations were used; in preparation of suspensions, 25 bulk drug substances and 10 industrially manufactured preparations were used; in preparation of topical solutions, 23 bulk drug substances and two industrially manufactured preparations were used; in preparation of topical powders, nine bulk drug substances were used; in preparation of oral solutions, five bulk drug substances were used. *Conclusions*: The analyzed prescriptions contained active ingredients used in Germany and the USA, as well as active ingredients, the use of which is limited in Germany and the USA. In Latvia, topical dosage forms containing two or more active ingredients are widely prescribed.

## 1. Introduction

Even though many industrially manufactured medicines are available, extemporaneous preparations still have their niche in dermatology. In several countries, dermatovenerologists are one of the specialists prescribing extemporaneous medicines the most [1,2,3].

However, extemporaneous compounding is one of the activities posing the highest risk in pharmacies [4]. Extemporaneous compounding errors may be committed at any stage starting from prescription of the medicine to issuing of the prepared medicine to the specific patient. One of the most dangerous errors involves incorrect calculations and microbiological contamination, which may result in a patient’s death. Cases were described when final compounded preparation missed the active ingredient or contained the incorrect active ingredient or excipients, or it was packed in incorrect packaging. It is important to mention that physicochemical issues, failure to follow instructions for preparation, or inappropriate preparation technology of extemporaneous medicines may result in undesirable precipitation or nonhomogeneous mixing [5,6].

Prior to compounding an extemporaneous prescription, the pharmacist should evaluate whether the compounded composition will be physically, chemically, and microbiologically stable during the entire shelf life [7]. Unavailability of proper research leads to additional threats relating to extemporaneous medicines, for example, formulation failure or exceeding or failure to reach a therapeutic concentration of active ingredients [4]. The number of active ingredients is important in the stability of the prepared dosage form. Extemporaneous medicines, in which several active ingredients are combined, are hard to evaluate, and it is hard to ensure the quality of the dosage form; therefore, it is recommended to combine two or more active ingredients only in justified exceptional cases [8,9].

In order to increase the quality of compounded medications and minimize risks to patient safety, several countries, for example, Germany and the United States of America (hereinafter referred to as USA), created standardized compounded preparation monographs. These monographs were developed based on rational justification in publications, and they guarantee the stability of prepared medicines during the specified shelf life, if the technology of preparation, indicated storage conditions, and correct packaging described in the monographs are used [10,11].

In Latvia, similarly to Germany and the USA, dermatovenerologists are one of the specialists prescribing extemporaneous medicines the most [12]. Despite that, Latvia, unlike these countries, does not have any officially approved standardized compounded preparation monographs for extemporaneous medicines [13]. Since monographs create a basis for prescription and preparation of safe and effective medicines, the creation of such monographs is important for Latvia. Prior to drafting compounded preparation monographs, it is essential to identify the current situation. Previous research provided data on the dosage form prescribed by dermatovenerologists the most [12]. The purpose of this survey is to analyze the extemporaneous prescriptions prescribed by Latvian dermatovenerologists to identify the active ingredients, combinations of active ingredients, and excipients prescribed by dermatovenerologists the most often, and to find out how many active ingredients are most often compounded in different dosage forms. To understand whether the extemporaneous formulations used in Latvia for dermatological indications are evidence-based, they were compared with German and USA formulations.

## 2. Materials and Methods

### 2.1. Dosage Forms, Active Ingredients, and Excipients in the Extemporaneous Prescriptions Prescribed by Latvian Dermatovenerologists

In accordance with Order No. 271 of the Cabinet of Ministers “On Statistical Regions of the Republic of Latvia and Administrative Units Included in Them”, Latvia is broken down into six statistical regions. Furthermore, the regions consist of republican cities and municipalities [14].

The pharmacies included in the research represented all six statistical regions, four republican cities, and seven municipalities. A database was created entering data on all the prescriptions prepared in the selected pharmacies in 2017 to summarize information on extemporaneous prescriptions.

The following data were summarized about each prescription:

- The pharmacy, in which the medicine was prepared;
- The statistical region and the republican city or municipality, where the pharmacy is located;
- The month, in which the prescription was prepared;
- The speciality of the healthcare professional, who prescribed the prescription, in accordance with the classifier of professions of healthcare professionals [15];
- The dosage form;
- All active ingredients;
- The number of active ingredients in the dosage form;
- All excipients.

All prescriptions prescribed by dermatovenerologists were selected for the research. Prescriptions prescribed by a specific physician could not be selected from the database, because identifying data were not entered. The exact number of dermatovenerologists is unknown, but the prescriptions represent all statistical regions of Latvia and provide an indication of the formulations prescribed by Latvian dermatovenerologists.

### 2.2. Comparisons of Latvian Extemporaneous Formulations with German and USA Formulations

In order to compare the extemporaneous formulations prescribed by Latvian dermatovenerologists with German and USA formulations, the sources containing compounded medication monographs as a standard of practice and quality in these countries were analyzed.

- Deutscher Arzneimittel-Codex/Neus Rezeptur-Formularium (hereinafter referred to as DAC/NRF);
- United States Pharmacopeia Compounding Compendium (hereinafter referred to as USP Compounding Compendium).

The German DAC/NRF was selected because it contains the biggest collection of standardized extemporaneous prescriptions in Europe, and more than half the prescriptions in it are intended for dermatological indications [7]. The USA USP Compounding Compendium was selected because it contains compounded preparation monographs, which are part of official text from the United States Pharmacopeia (USP) National Formulary (NF) [16].

The active ingredients, combinations of active ingredients, and excipients prescribed by Latvian dermatovenerologists were searched in these sources according to the dosage form.

If the DAC/NRF and USP Compounding Compendium did not contain any of the active ingredients prescribed in Latvia, then they were searched in German and USA professional literature on extemporaneous compounding.

### 2.3. Ethical Approval

The study “Availability of extemporaneous preparations in Latvian pharmacies: a quantitative and qualitative assessment of the situation and prospect for the future” was allowed by the Ethics Committee of Riga Stradins University (Identification code Nr. 14, date of approval 5 October 2017).

## 3. Results

Data from 17 Latvian pharmacies were collected. In total, 2521 extemporaneous formulations were prepared in these pharmacies based on prescriptions issued by dermatovenerologists in 2017. Dermatovenerologists prescribed semi-solid dosage forms, suspensions, and topical and oral solutions, as well as topical powders, an oral powder, and nasal drops. An oral powder and nasal drops were prescribed once and, therefore, were not analyzed in this study.

### 3.1. Number of Active Ingredients in the Dosage Forms Prescribed by Dermatovenerologists

Bulk drug substances and industrially manufactured finished dosage forms containing active ingredients—oral capsules, tablets, solutions, ointments, and creams—used instead of bulk drug substances are classified as active ingredients in this article. More than half of the semi-solid dosage forms (77.71%), suspensions (98.54%), topical solutions (51.24%), and topical powders (97.92%) contained two or more active ingredients, while oral solutions most often contained one active ingredient (62.92%). In several analyzed prescriptions, physicians prescribed only the base without active ingredients, for example, ointment prepared from vegetable oil, purified water, and wool fat (Table 1, Table 2, Table 3, Table 4 and Table 5).

### 3.2. Semi-Solid Dosage Forms

#### 3.2.1. Active Ingredients in Semi-Solid Dosage Forms

In total, 25 bulk drug substances and 37 industrially manufactured preparations were used in the preparation of semi-solid dosage forms. Table 6 includes those active ingredients, which were prescribed in 10 or more prescriptions (Table 6). Table 6 does not include industrially manufactured creams and ointments, because they are viewed in detail in Table 7.

Industrially manufactured dosage forms—ointments and creams—were widely prescribed in the preparation of extemporaneous semi-solid dosage forms. Dermatovenerologists most frequently used creams and ointments containing corticosteroids, antifungal agents (imidazole and triazole derivatives), and their combinations (Table 7).

#### 3.2.2. Characteristics of Active Ingredients Most Frequently Prescribed in Preparation of Semi-Solid Dosage Forms, Their Most Common Combinations, and Comparison with German and USA Formulations

The ingredients described in this section and their prescription frequency are shown in Table 6.

Depending on the concentration, salicylic acid produces a pronounced keratolytic effect, as well as anti-inflammatory, antifungal, and antibacterial action. Salicylic acid is mentioned in the DAC/NRF in the composition of several ointments, creams, and pastes. In these formulations, salicylic acid is used as the only active ingredient or in combination with dithranol or solution of coal tar in ethanol 96% (*v*/*v*) and polysorbate 80 [10]. In Latvia, salicylic acid was prescribed as the only active ingredient in 72 prescriptions. USP Compounding Compendium describes the preparation of salicylic acid–zinc oxide paste [11]. In Latvia, such a combination was found in 56 of the analyzed prescriptions. In the prescriptions issued by Latvian dermatologists and prepared in pharmacies, salicylic acid was most frequently combined with sulfur (424 prescriptions), isoconazole nitrate and diflucortolone valerate cream (72 prescriptions), prednisolone tablets (66 prescriptions), and birch tar (60 prescriptions). Such combinations can neither be found in the DAC/NRF nor in the USP Compounding Compendium [10,11].

Sulfur demonstrates antiparasitic, keratolytic, and antiseptic action [17]. The DAC/NRF does not contain formulations with sulfur. In 1996, an ointment containing it was excluded from the DAC/NRF due to a negative sulfur use benefit-and-risk assessment [10]. Prescription of sulfur in dermatological prescriptions is permitted with the evaluation of risks and benefits, and prescriptions with sulfur can be found in German dermatological literature [17,18]. The USP Compounding Compendium includes sulfur ointment, where sulfur is the only active ingredient [11]. In Latvia, sulfur was prescribed as the only active ingredient in three prescriptions. In other analyzed prescriptions of Latvian dermatovenerologists, sulfur was most frequently combined with the same active ingredients with which salicylic acid was combined. Sulfur together with salicylic acid was prescribed in 424 prescriptions, with isoconazole nitrate and diflucortolone valerate cream in 73 prescriptions, with prednisolone tablets in 50 prescriptions, with birch tar in 47 prescriptions, and with zinc oxide in 43 prescriptions.

Prednisolone demonstrates anti-inflammatory and antipruritic action [17]. The DAC/NRF contains creams with prednisolone prodrug prednicarbate and prednisolone acetate. In these formulations, prednisolone acetate is used as the only active ingredient, but prednicarbate is used alone and in combination with octenidine hydrochloride [10]. Furthermore, prednisolone cream prescription can be found in German dermatological literature as a cheaper alternative for industrially manufactured prednisolone creams [19]. The USP Compounding Compendium does not contain semi-solid dosage forms with prednisolone [11]. Unlike in Germany, analyzed Latvian prescriptions used prednisolone tablets rather than bulk drug substance for the preparation of semi-solid dosage forms. In Latvia, prednisolone was prescribed as the only active ingredient in eight prescriptions. In the analyzed prescriptions of Latvian pharmacies, prednisolone was most often combined with the following active ingredients: salicylic acid (66 prescriptions); ampicillin trihydrate capsules (55 prescriptions); sulfur (50 prescriptions); zinc oxide (41 prescriptions); boric acid (39 prescriptions). Such combinations were not included in the DAC/NRF [10].

Zinc oxide produces a desiccating action, as well as a mild anti-inflammatory and tightening action [17]. Zinc oxide is mentioned in the DAC/NRF in the composition of several pastes, in addition to as a liniment. In these formulations, zinc oxide is used as the only active ingredient or in combination with the following active ingredients: dithranol, ichthammol, and bismuth subgallate [10]. The USP Compounding Compendium includes ointment containing zinc oxide and pastes, where zinc oxide is the only active ingredient or in combination with salicylic acid [11]. In Latvia, zinc oxide was prescribed as the only active ingredient in two prescriptions. In the analyzed prescriptions of Latvian pharmacies, zinc oxide was most often combined with the following active ingredients: salicylic acid (56 prescriptions); ichthammol (47 prescriptions); sulfur (43 prescriptions); prednisolone tablets (41 prescriptions); birch tar (32 prednisolone); resorcinol (32 prescriptions); bismuth subgallate (30 prescriptions). Out of these combinations in the DAC/NRF and USP Compounding Compendium, none can be found of zinc oxide with sulfur, prednisolone, and birch tar [10,11].

Metronidazole has antibacterial, antiparasitic, and anti-inflammatory properties [17]. The DAC/NRF contains creams and a gel containing metronidazole as the only active ingredient and in combination with erythromycin [10]. Such a combination can also be found in the analyzed Latvian prescriptions. Metronidazole was prescribed as the only active ingredient in 65 analyzed Latvian prescriptions. The USP Compounding Compendium does not include semi-solid dosage forms with metronidazole [11]. Metronidazole tablets rather than bulk drug substance were mainly used for the preparation of semi-solid dosage forms in the analyzed Latvian prescriptions. Latvian dermatovenerologists most frequently combined metronidazole with the following active ingredients: sulfur (31 prescriptions); salicylic acid (27 prescriptions); clotrimazole cream (20 prescriptions); isoconazole nitrate and diflucortolone valerate cream (13 prescriptions); erythromycin tablets (13 prescriptions). As already mentioned, out of these combinations, only a metronidazole combination with erythromycin can be found in the DAC/NRF.

Dexamethasone from the first class of steroids demonstrates anti-inflammatory and antipruritic action [17]. The DAC/NRF describes only the preparation of dexamethasone 1% and 10% trituration, but the formulations containing dexamethasone are not included in this formulary [10]. Ointments, creams, and hydrogels containing dexamethasone can be found in German dermatological literature [17,18]. The USP Compounding Compendium does not include semi-solid dosage forms with dexamethasone [11]. Dexamethasone tablets rather than bulk drug substance were used for the preparation of semi-solid dosage forms in the analyzed prescriptions of Latvian pharmacies. Dexamethasone was prescribed as the only active ingredient in six prescriptions. In the analyzed prescriptions of Latvian pharmacies, dexamethasone was most frequently combined with the following active ingredients: salicylic acid (43 prescriptions); fluocinolone acetonide ointment (22 prescriptions); benzocaine (21 prescriptions); birch tar (21 prescriptions); sulfur (17 prescriptions).

Birch tar is not included in the DAC/NRF, German dermatological literature, or the USP Compounding Compendium [10,11,17,18,19]. The DAC/NRF includes coal tar, which is used in the preparation of ointments and creams. In the DAC/NRF, coal tar is not combined with other active ingredients, but is used as the only active ingredient [10]. The USP Compounding Compendium also uses coal tar, but in combination with zinc oxide in the respective ointments [11]. In the analyzed prescriptions of Latvian pharmacies, birch tar was prescribed, which demonstrates a disinfecting and local irritant action [20]. In these prescriptions, birch tar was most frequently combined with the following active ingredients: salicylic acid (60 prescriptions); sulfur (47 prescriptions); zinc oxide (32 prescriptions); turpentine oil (30 prescriptions); dexamethasone tablets (21 prescriptions). Birch tar was prescribed as the only active ingredient in three prescriptions.

In Germany, the use of boric acid and its salts for the preparation of semi-solid dosage forms is prohibited due to low efficacy and risk of resorptive poisoning. Boric acid can be used only in the preparation of homeopathic preparations, as well as in the preparation of eye drops, where it is used as a buffer [10]. The USP Compounding Compendium also does not include semi-solid dosage forms with boric acid [11]. Latvian dermatologists still prescribe boric acid, most probably as an antiseptic; in the analyzed prescriptions, the most common combinations were with prednisolone tablets (39 prescriptions), ampicillin trihydrate capsules (36 prescriptions), and zinc oxide (13 prescriptions).

In 1999, mercuric oxide was removed from the DAC/NRF [10]. It is also not included in the USP Compounding Compendium [11]. In Latvia, mercuric oxide is still prescribed as an antiseptic and as the only active ingredient (38 prescriptions), but it is also found in combinations. The most common combinations were with the following active ingredients: salicylic acid (16 prescriptions); prednisolone tablets (nine prescriptions).

Ampicillin is an antibiotic from the group of broad-spectrum penicillins [21]. Formulations with ampicillin are not included in the DAC/NRF and USP Compounding Compendium [10,11]. Latvian prescriptions include ampicillin trihydrate oral capsules rather than bulk drug substance; the most common combinations were with prednisolone tablets (55 prescriptions), boric acid (36 prescriptions), ichthammol (17 prescriptions), and zinc oxide (16 prescriptions). Ampicillin was not used as the only active ingredient in semi-solid dosage forms.

Diphenhydramine hydrochloride is a histamine H1 antagonist [22]. The DAC/NRF and USP Compounding Compendium do not contain formulations with this active ingredient [10,11]. In Germany, the antipruritic and antihistamine effect of locally used diphenhydramine hydrochloride is subject to discussion, and its use is also limited due to the risk of potential sensibilization [23]. At the same time, there are industrially manufactured medicines—gels with diphenhydramine hydrochloride [24,25]. USA literature mentions extemporaneously compounded gels containing diphenhydramine hydrochloride [6]. In Latvia, diphenhydramine hydrochloride was not used as the only active ingredient in the preparation of semi-solid dosage forms; it was most frequently combined with the following active ingredients: sulfur (34 prescriptions); salicylic acid (32 prescriptions); procaine hydrochloride (15 prescriptions); dexamethasone tablets (14 prescriptions).

Ichthammol demonstrates anti-inflammatory and antiseptic action [17]. The DAC/NRF contains a cream and a paste containing ichthammol, where ichthammol is in combination with zinc oxide [10]. Such a combination can also be found in the analyzed Latvian prescriptions. The USP Compounding Compendium contains ichthammol ointment, where ichthammol is the only active ingredient [11]. In Latvia, ichthammol was prescribed as the only active ingredient in one prescription. In the analyzed prescriptions of Latvian pharmacies, ichthammol was most often combined with the following active ingredients: zinc oxide (47 prescriptions); prednisolone tablets (37 prescriptions); resorcinol (32 prescriptions); ampicillin trihydrate capsules (17 prescriptions); salicylic acid (12 prescriptions).

Benzocaine demonstrates anesthetic and analgesic action [17]. The DAC/NRF and USP Compounding Compendium do not contain formulations with benzocaine [10,11]. German dermatological literature describes benzocaine ointments, creams, and gels [17,18]. In Latvia, benzocaine was prescribed as the only active ingredient in one prescription. In the analyzed prescriptions of Latvian pharmacies, benzocaine was most frequently combined with the following active ingredients: dexamethasone tablets (21 prescriptions); salicylic acid (16 prescriptions); fluocinolone acetonide ointment (12 prescriptions); prednisolone tablets (10 prescriptions).

Bismuth subgallate demonstrates tightening and antiseptic properties [17]. As described previously, the DAC/NRF includes a paste which contains a combination of bismuth subgallate and zinc oxide [10]. Also, in the analyzed Latvian prescriptions, bismuth subgallate was most frequently combined with zinc oxide (30 prescriptions). The USP Compounding Compendium does not contain semi-solid dosage forms with bismuth subgallate [11].

Resorcinol demonstrates keratolytic and antiseptic action [26]. All formulations with resorcinol were removed from the DAC/NRF in 1996 [10]. In Germany, the use of resorcinol in dermatology is subject to discussion. Alternatives with a better risk–benefit ratio are offered. For example, chlorhexidine gluconate may be used as an antiseptic, but salicylic acid is offered for a keratolytic effect [26]. The USP Compounding Compendium does not contain semi-solid dosage forms with resorcinol for use in dermatology [11]. In the analyzed prescriptions of Latvian pharmacies, resorcinol was most frequently prescribed in the following combination: resorcinol–prednisolone tablets–zinc oxide–ichthammol (32 prescriptions); 12 of these prescriptions also contained ampicillin trihydrate capsules.

Lactic acid has antiseptic, keratolytic, and escharotic action [17]. In the DAC/NRF, lactic acid is mentioned as an excipient for stabilization of pH for a cream with urea [10]. German dermatological literature describes ointments and creams where lactic acid is an active ingredient [17,18]. The USP Compounding Compendium does not contain semi-solid dosage forms with lactic acid [11], but USA literature contains an ointment where the function of lactic acid is to increase hydration of the skin. In this ointment, lactic acid was combined with urea and triamcinolone acetonide [6]. In the analyzed prescriptions of Latvian pharmacies, lactic acid was most frequently combined with salicylic acid (32 prescriptions). In total, 26 prescriptions included only these two active ingredients, but five prescriptions also contained sulfur, and one prescription contained ichthammol along with this combination. Lactic acid was prescribed as the only active ingredient in one prescription.

Turpentine oil demonstrates local irritant, analgesic, and antiseptic action [20]. The DAC/NRF does not contain semi-solid dosage forms with turpentine oil used in dermatology [10]. The USP Compounding Compendium does not include semi-solid dosage forms with turpentine oil [11]. In the analyzed prescriptions of Latvian pharmacies, turpentine oil was most often prescribed in two main combinations: turpentine oil–salicylic acid–birch tar–sulfur–zinc oxide (17 prescriptions) and turpentine oil–salicylic acid–birch tar–sulfur–zinc oxide–benzocaine (12 prescriptions). In Latvia, an ointment containing turpentine oil is also produced industrially.

Procaine hydrochloride is an anesthetic and analgesic agent [17]. The DAC/NRF does not contain formulations with this active ingredient, but German dermatological literature describes ointments, creams, and hydrogels with it [10,17]. The USP Compounding Compendium does not contain semi-solid dosage forms with procaine hydrochloride [11]. In the analyzed prescriptions of Latvian pharmacies, procaine hydrochloride was most frequently combined with salicylic acid (27 prescriptions) and sulfur (26 prescriptions).

Erythromycin has bacteriostatic and, in large doses, bactericide action [17]. The DAC/NRF contains creams and gels with erythromycin as the only active ingredient or, as described above, in combination with metronidazole. It is emphasized in the DAC/NRF that the dosage forms with erythromycin should only be prepared based on standardized prescriptions, because erythromycin has low stability depending on the pH environment [10]. The USP Compounding Compendium does not contain semi-solid dosage forms with erythromycin [11]. Oral erythromycin tablets rather than bulk drug substance were used for the preparation of semi-solid dosage forms in the analyzed prescriptions of Latvian pharmacies. In these prescriptions, erythromycin was most frequently combined with metronidazole (13 prescriptions). In one prescription, erythromycin was combined with a boric acid, lactic acid, zinc oxide, and fluocinolone acetonide ointment. Erythromycin was prescribed as the only active ingredient in four prescriptions.

Menthol demonstrates hyperemic, antirheumatic, analgesic, and antipruritic action [17]. The DAC/NRF contains ointments and cream with menthol. In these formulations, menthol is used as the only active ingredient or in combinations, which are not used in dermatology, such as menthol–camphor–methyl salicylate and menthol–camphor–turpentine oil–eucalypt oil–mountain pine oil [10]. The USP Compounding Compendium does not contain semi-solid dosage forms with menthol, but USA literature describes the preparation of a testosterone–menthol eutectic ointment, as well as the preparation of a lip balm containing menthol [5,11]. In the analyzed prescriptions of Latvian pharmacies, the most frequent combination of active ingredients was menthol–diphenhydramine hydrochloride–boric acid (six prescriptions). Industrially manufactured creams or ointments with corticosteroids, and corticosteroids together with antibiotics or antifungal agents were added to this combination.

#### 3.2.3. Excipients Used in Preparation of Semi-Solid Dosage Forms and Comparison with German and USA Formulations

In total, 14 excipients were used in the analyzed prescriptions prescribed by Latvian dermatovenerologists (Table 8), but the prescriptions also included excipients from finished industrially manufactured dosage forms.

The following excipients were prescribed the most often: soft paraffin, wool fat, purified water, sunflower oil, potato starch, Wolff Basis Creme or Basiscreme DAC, olive oil, and liquid paraffin. These excipients were searched in German and USA professional literature.

The formulations of dermatological semi-solid dosage forms in the DAC/NRF contain excipients used in Latvia such as soft paraffin, liquid paraffin, wool fat, purified water, starch, and Basiscreme DAC. Sunflower oil and olive oil cannot be found in the composition of semi-solid dosage forms included in the DAC/NRF. However, a paste formulation is described, where soft paraffin together with another vegetable oil (flax seed oil) is used as a base. Wolff Basis Creme is also not included in the DAC/NRF, but there are standardized formulations with this cream that were created by the manufacturer. The formulations included in the DAC/NRF, along with the traditional bases used in Latvia, also use other bases; for example, wool alcohols are used as absorption bases and macrogols are used as water-soluble bases, while Nichtionische hydrophile Creme SR (Standardisierte Rezepturen) and Anionische hydrophile Creme SR are used as emulsifying bases [10].

Semi-solid dosage formulations in the USP Compounding Compendium, as in the DAC/NRF, contain soft paraffin, liquid paraffin, wool fat, purified water, and starch. Sunflower oil, olive oil, and Wolff Basis Creme are not included in the compositions of semi-solid dosage forms available in the USP Compounding Compendium. Similarly to Germany, the USP Compounding Compendium also uses other bases; for example, white ointment USP is used as a hydrocarbon base, hydrophilic petrolatum USP is offered as an anhydrous absorption base, and hydrophilic ointment USP is used as a water-removable base, while polyethylene glycol ointment NF is used as a water-soluble base [6,11]. No such bases are prescribed in the analyzed Latvian prescriptions.

### 3.3. Suspensions

#### 3.3.1. Active Ingredients Used in Preparation of Suspensions

In total, 25 bulk drug substances and 10 industrially manufactured preparations were used in preparation of suspensions. Table 9 includes those active ingredients which were prescribed in 10 or more prescriptions (Table 9).

#### 3.3.2. Characteristics of Active Ingredients Most Frequently Used in Preparation of Suspensions, Their Most Common Combinations, and Comparison with German and USA Formulations

As already mentioned above, it is allowed to use boric acid and its salts for preparation of medicines in Germany only in individual cases, for example, as a buffer in eye drops. It is prohibited to prescribe boric acid in the composition of suspensions used in dermatology [10]. The USP Compounding Compendium also does not mention boric acid in suspensions; it is mentioned only as a potential stabilizer in the composition of aluminum subacetate topical solution [11]. Similarly to Germany, USA extemporaneous literature also mentions boric acid as an excipient in several eye drops [5,6]. In Latvia, however, boric acid is prescribed as a disinfectant [27,28]. In the analyzed prescriptions of Latvian pharmacies, boric acid was most often combined with the following active ingredients: salicylic acid (561 prescriptions); sulfur (459 prescriptions); camphor (385 prescriptions); sulfathiazole (119 prescriptions); chloramphenicol (74 prescriptions); resorcinol (64 prescriptions).

The properties of salicylic acid, leading to its use in dermatology, were described above. The DAC/NRF and USP Compounding Compendium do not contain suspensions with this active ingredient [10,11]. In Latvia, salicylic acid as the only active ingredient was prescribed in two of the analyzed prescriptions, where its solubility limit in olive oil was exceeded and suspensions rather than solutions were prepared. In the prescriptions of suspensions prescribed by Latvian dermatovenerologists, most common combinations of salicylic acid were identical to the previously described combinations of boric acid: boric acid (561 prescriptions); sulfur (450 prescriptions); camphor (356 prescriptions); sulfathiazole (111 prescriptions); resorcinol (62 prescriptions); chloramphenicol (48 prescriptions).

As already mentioned above, the DAC/NRF does not contain formulations containing sulfur [10]. However, German dermatological literature includes suspensions with sulfur. For example, a suspension for use on skin is mentioned, where sulfur is combined with zinc oxide [29]. In Latvia, such a combination was found in 21 of the analyzed prescriptions. Out of these prescriptions, two contained only the said two active ingredients, but another 19 prescriptions had 1–4 active ingredients added to the combination of sulfur and zinc oxide. The USP Compounding Compendium, similarly to DAC/NRF, does not contain suspensions with sulfur, but USA extemporaneous literature describes a suspension containing sulfur for use on skin [6,11]. In the analyzed prescriptions of Latvian pharmacies, sulfur was most often combined with the following active ingredients: boric acid (459 prescriptions); salicylic acid (450 prescriptions); camphor (355 prescriptions); sulfanilamide (52 prescriptions); chloramphenicol (48 prescriptions).

Camphor demonstrates anti-inflammatory and antipruritic action [17]. The DAC/NRF does not include liquid dosage forms containing camphor [10]. In the analyzed suspension prescriptions of Latvian pharmacies, camphor was most frequently used as an alcohol solution. A standardized formulation of camphor alcohol is also available in the German Pharmacopoeia [30]. The USP Compounding Compendium describes the preparation of camphor alcohol [11]. In the analyzed prescriptions of Latvian pharmacies, camphor alcohol was most often combined with the following active ingredients: boric acid (385 prescriptions); salicylic acid (356 prescriptions); sulfur (355 prescriptions); chloramphenicol (34 prescriptions); sulfanilamide (30 prescriptions).

Sulfathiazole is an antibacterial agent of the sulfonamide group [20]. Since 1991, the sulfathiazole monograph was removed from the DAC/NRF [10]. In Germany, the use of sulfonamide group preparations on the skin is considered to be unjustified, except for individual cases. The reason for that is the low antimicrobial activity of sulfonamide group preparations and high risk of sensibilization [31]. The USP Compounding Compendium also does not contain formulations with this ingredient [11]. In the analyzed prescriptions of Latvian pharmacies, sulfathiazole was most often combined with the following active ingredients: boric acid (119 prescriptions); salicylic acid (111 prescriptions); sulfur (15 prescriptions); camphor (nine prescriptions); calendula tincture (eight prescriptions).

Thanks to its mild antimicrobial and anti-inflammatory action, as well as tightening and wound-healing properties, zinc oxide is included in the composition of several suspensions in the DAC/NRF. Zinc oxide is used in them as the only active ingredient or in combination with ichthammol, solution of coal tar in quillaia bark tincture and ethanol 70% (*v*/*v*), Lauromacrogol 400, and nystatin [10]. In Latvia, zinc oxide as the only active ingredient was prescribed in nine prescriptions, but zinc oxide in combination with ichthammol was found in one of the analyzed prescriptions. The USP Compounding Compendium describes preparation of a suspension containing zinc oxide, where zinc oxide is combined with calamine [11]. In the prescriptions prescribed by Latvian dermatovenerologists, zinc oxide was most often combined with menthol (47 prescriptions), diphenhydramine hydrochloride (45 prescriptions), boric acid (39 prescriptions), sulfur (21 prescriptions), and benzocaine (16 prescriptions).

Chloramphenicol demonstrates bacteriostatic action against Gram-positive and Gram-negative pathogenic microorganisms [17]. Due to the high risk of sensibilization, the DAC/NRF does not include chloramphenicol suspensions for use in dermatology [10]. The DAC/NRF information source describes a suspension for use on skin, where chloramphenicol is combined with zinc oxide, but its use is permissible only in exceptional cases [32]. In Latvia, such a combination is only found in two of the analyzed suspension prescriptions. The USP Compounding Compendium does not contain formulations with this ingredient for use in dermatology [11]. In the analyzed prescriptions of Latvian pharmacies, chloramphenicol was most often combined with boric acid (74 prescriptions), salicylic acid (48 prescriptions), sulfur (48 prescriptions), and camphor (34 prescriptions).

Despite the fact that all the formulations containing resorcinol were removed from the DAC/NRF (see Section 3.2.2, resorcinol), in the cooperation project between physicians and pharmacists, a suspension formulation for use in dermatology, where resorcinol is combined with salicylic acid and sulfur, is mentioned [33]. In Latvia, such a combination was found in 26 of the analyzed suspension prescriptions. The USP Compounding Compendium does not contain suspensions with resorcinol [11]. In the prescriptions prescribed by Latvian dermatovenerologists, resorcinol was most often combined with boric acid (64 prescriptions), salicylic acid (62 prescriptions), sulfur (31 prescriptions), and camphor (25 prescriptions).

Although the DAC/NRF does not contain suspensions with menthol, German dermatological literature considers the possibility of adding menthol to the DAC/NRF suspensions containing zinc oxide [10,34]. It is similar in the USA; the USP Compounding Compendium also does not contain suspensions with menthol [11], but USA dermatological literature mentions suspension formulations containing menthol. For example, there is a combination of menthol–hydrocortisone–calamine, where menthol is used in prescription as an antipruritic and local analgesic [6]. In the analyzed prescriptions of Latvian pharmacies, menthol was most often combined with zinc oxide (47 prescriptions), boric acid (26 prescriptions), diphenhydramine hydrochloride (17 prescriptions), and benzocaine (15 prescriptions).

Sulfanilamide, similarly to sulfathiazole, belongs to the sulfonamide group. Sulfanilamide demonstrates bacteriostatic properties against Gram-positive and Gram-negative bacteria [35]. In the DAC/NRF, sulfanilamide is mentioned only as a reagent for the preparation of control solutions [10]. The potential reason for the non-existence of formulations with active ingredients from the sulfonamide group is described above (see Section 3.3.2, sulfathiazole). The USP Compounding Compendium also does not contain formulations with sulfanilamide [11]. In Latvia, sulfanilamide is still being prescribed. In the analyzed prescriptions of Latvian pharmacies, sulfanilamide was not prescribed as the only active ingredient; in all prescriptions, it was used in combinations with boric acid. An additional 1–5 other active ingredients were added to this combination, most frequently sulfur (52 prescriptions) and salicylic acid (44 prescriptions).

The DAC/NRF and USP Compounding Compendium do not contain suspensions with histamine H1 antagonist diphenhydramine hydrochloride [10,11]. However, dermatovenerologists in Latvia still prescribe this active ingredient, diphenhydramine hydrochloride was most frequently prescribed in combination with zinc oxide (45 prescriptions).

The DAC/NRF and USP Compounding Compendium do not contain suspensions with acetic acid [10,11]. However, it is mentioned in these sources, as well as in German and USA dermatological literature in relation to the preparation of topical solutions (see Section 3.4.2, acetic acid). In the analyzed prescriptions of Latvian pharmacies, acetic acid was most often combined with the following active ingredients: sulfur (20 prescriptions); salicylic acid (18 prescriptions); iodine tincture 5% (13 prescriptions); dimethyl sulfoxide (13 prescriptions); terbinafine hydrochloride tablets (13 prescriptions).

In Latvia, calendula tincture is produced industrially; according to the manufacturer, it is used for the treatment of small wounds and skin inflammation [36]. The DAC/NRF and USP Compounding Compendium do not contain suspensions with calendula tincture [10,11]. In the analyzed prescriptions of Latvian pharmacies, calendula tincture was most often combined with the following active ingredients: boric acid (32 prescriptions); salicylic acid (26 prescriptions); sulfur (25 prescriptions); metronidazole tablets (14 prescriptions).

The DAC/NRF and USP Compounding Compendium do not contain suspensions with metronidazole for use in dermatology [10,11]. However, German dermatological literature mentions several industrially manufactured bases, which can be used to prepare metronidazole lotion [18]. In the analyzed Latvian prescriptions, preparation of suspensions mainly used metronidazole tablets, which, in accordance with the information provided in the summary of product characteristics, should be used orally for the treatment and prophylaxis of infections caused by microorganisms sensitive to metronidazole [37]. Latvian dermatovenerologists prescribed metronidazole in combination with boric acid in all the analyzed prescriptions for suspensions. Out of these prescriptions, two only contained the said two active ingredients, but another 21 prescriptions had 2–5 active ingredients added to the combination of metronidazole and boric acid, for example, salicylic acid (19 prescriptions), sulfur (15 prescriptions), and calendula tincture (14 prescriptions).

Suspensions with lactic acid are also not included in the DAC/NRF and USP Compounding Compendium [10,11]. In the analyzed prescriptions of Latvian pharmacies, lactic acid was most often combined with boric acid and salicylic acid (12 prescriptions), while another 1–4 active ingredients were added to this combination.

The DAC/NRF and USP Compounding Compendium do not contain suspensions with benzocaine for use in dermatology [10,11], but USA literature describes the preparation of an antiseptic and anesthetic solution, where benzocaine is combined with benzethonium chloride [6]. In the analyzed prescriptions of Latvian pharmacies, benzocaine was most frequently combined with the following active ingredients: zinc oxide (16 prescriptions); menthol (15 prescriptions).

#### 3.3.3. Excipients Used in Preparation of Suspensions and Comparison with German and USA Formulations

In total, 12 excipients were used in the prescriptions prescribed by Latvian dermatovenerologists (Table 10), but the prescriptions also included excipients from finished industrially manufactured dosage forms used in the production of extemporaneous medicines.

The following excipients were prescribed the most often: purified water, ethanol, glycerol, and talc. These excipients were searched in German and USA professional literature.

The DAC/NRF contains all the above mentioned excipients in suspension formulations for use in dermatology. Unlike in Latvia, iron oxides used as color pigments were added to several suspensions containing zinc oxide [10]. The USP Compounding Compendium contains a calamine topical suspension formulation with excipients, which are also used in Latvia with water and glycerol [11].

### 3.4. Topical Solutions

#### 3.4.1. Active Ingredients Used in Preparation of Topical Solutions

In total, 23 bulk drug substances, as well as two industrially manufactured preparations (calendula tincture and iodine tincture 5%), were used in preparation of topical solutions. Table 11 includes those active ingredients which were prescribed in 10 or more prescriptions (Table 11).

#### 3.4.2. Characteristics of Active Ingredients Most Frequently Used in Preparation of Topical Solutions, Their Most Common Combinations, and Comparison with German and USA Formulations

The DAC/NRF does not contain prescriptions with acetic acid for use in dermatology; there is only one formulation of ear drops, which contains acetic acid as the only active ingredient. These ear drops demonstrate drying, bactericide, and fungicide action [10]. However, acetic acid solutions of different concentrations, which are used as an antiseptic, can be found in German dermatological literature [38]. The USP Compounding Compendium includes a preparation monograph for diluted acetic acid [11]. USA extemporaneous literature also includes a solution containing acetic acid, which is used as a wart remover. Acetic acid in this prescription is combined with lactic acid and salicylic acid, but a flexible collodion is used as a vehicle [6]. In Latvia, acetic acid was prescribed both as the only active ingredient (86 prescriptions) and in combinations (116 prescriptions). In the analyzed prescriptions, acetic acid was most often prescribed in the following three main combinations: acetic acid–salicylic acid (43 prescriptions), acetic acid–iodine (39 prescriptions), and acetic acid–salicylic acid–iodine (25 prescriptions).

The use of boric acid and its salts in liquid dosage forms in Germany and the USA was already described in the section of suspensions. Contrary to Germany and the USA, where boric acid is not used as an active ingredient, Latvian dermatovenerologists still prescribe topical solutions containing boric acid. Boric acid was prescribed as the only active ingredient in 59 prescriptions for topical solutions, while the main combinations were as follows: boric acid–liquefied phenol–resorcinol (14 prescriptions) and boric acid–liquefied phenol–resorcinol–fuchsin (15 prescriptions); other components, which were added to these combinations, were acetone, ethanol, and purified water. In Latvia, these compositions are named “Castellani solution” and “Castellani solution, colorless”. “Castellani solution” and “Castellani solution, colorless” were also prepared in Germany. As a result of research in Germany, the compositions of these solutions were improved and partially changed, but their names remained unchanged. For example, before 1983, Germany prepared the compositions currently used in Latvia; however, in 1983–1996, these compositions were prepared without boric acid, and phenol was replaced with chlorocresol. Today, Germany offers chlorhexidine alcoholic solution as a therapeutic alternative to “Castellani solution” [39,40].

Salicylic acid in the DAC/NRF is included in the composition of several topical solutions. In these formulations, it is the only active ingredient, or in combination with lactic acid or triamcinolone acetonide [10]. In Latvia, salicylic acid was prescribed as the only active ingredient in 11 prescriptions for topical solutions. In the USP Compounding Compendium, salicylic acid is used in combination with a flexible collodion [11]. In the analyzed prescriptions of Latvian pharmacies, salicylic acid was most frequently combined with acetic acid (69 prescriptions) and iodine (31 prescriptions).

Iodine demonstrates antiseptic and disinfecting action [41]. The DAC/NRF describes the preparation of iodine water and glycerol solutions in different concentrations; however, they are not intended for dermatological indications [10]. The USP Compounding Compendium provides several formulations of solutions with iodine with different environments [11]. In Latvia, iodine as the only active ingredient was prescribed in 16 prescriptions; in other prescriptions it was combined with one or two other active ingredients as follows: iodine–acetic acid (39 prescriptions); iodine–salicylic acid (6 prescriptions); iodine–acetic acid–salicylic acid (25 prescriptions); iodine–resorcinol–benzoic acid (30 prescriptions). Industrially manufactured iodine tincture 5% in combination with acetic acid was prescribed in four prescriptions.

As already mentioned above, the formulations containing resorcinol were removed from the DAC/NRF. Before that, the formulary contained a formulation of topical solution, where resorcinol was in combination with salicylic acid, as well as formulations of Castellani solutions [26,40]. The USP Compounding Compendium describes the preparation of carbol–fuchsin topical solution, where resorcinol is combined with basic fuchsin and phenol [11]. The analyzed prescriptions of Latvian pharmacies also contained solutions with such a combination (15 prescriptions); however, unlike the USP Compounding Compendium, boric acid was added to the combination. Resorcinol was prescribed as the only active ingredient in Latvia in 14 prescriptions.

In an acidic environment, benzoic acid has bacteriostatic and fungistatic properties [42]. The DAC/NRF and USP Compounding Compendium do not contain solutions for use in dermatology with benzoic acid as an active ingredient [10,11]. However, German literature mentions a formulation with antifungal indications, where benzoic acid was combined with salicylic acid [42]. In Latvia, such a combination was found in 21 of the analyzed prescriptions for topical solutions; in these prescriptions, 1–2 other active ingredients were added to the combination, most frequently boric acid (19 prescriptions). The most common combination was benzoic acid–resorcinol–iodine (30 prescriptions).

Phenol demonstrates antipruritic, antiseptic, and topical anesthetic properties [6]. The DAC/NRF does not contain dosage forms with phenol for use in dermatology. In Germany, phenol as an active ingredient is not used on skin and mucous membranes, with the exception of individual cases, when it is used only once or in small amounts [10]. The USP Compounding Compendium includes the already mentioned carbol–fuchsin topical solution, as well as phenolated calamine topical suspension, where liquefied phenol is combined with calamine and zinc oxide [11]. Formulations with it for use in dermatology can also be found in USA literature. For example, a suspension exists where phenol is combined with zinc sulfate and sulfurated potash [6]. In the analyzed prescriptions of Latvian pharmacies, phenol was prescribed only in the composition of Castellani solution.

The DAC/NRF contains only one eye-drop formulation with chloramphenicol [10]. The DAC/NRF information source describes topical solutions containing chloramphenicol, but with an indication that the use of chloramphenicol on the skin is considered outdated and should be used only in exceptional cases [43,44]. The USP Compounding Compendium also does not contain solutions with this active ingredient [11]. In Latvia, chloramphenicol was not used as the only active ingredient in preparation of topical solutions; instead, it was prescribed in the following combinations: chloramphenicol–boric acid (11 prescriptions) and chloramphenicol–benzocaine (five prescriptions).

Fuchsin demonstrates antiseptic and antimycotic action [45]. At present, the DAC/NRF does not contain formulations with fuchsin [10]. As already mentioned above, the USP Compounding Compendium includes a topical solution with basic fuchsin [11]. In the analyzed prescriptions of Latvian pharmacies, fuchsin was prescribed only in the composition of Castellani solution (15 prescriptions).

#### 3.4.3. Excipients Used in Preparation of Topical Solutions and Comparison with German and USA Formulations

In the analyzed prescriptions of Latvian dermatovenerologists, 11 excipients were used for the preparation of topical solutions (Table 12).

Purified water, ethanol, glycerol, potassium iodide (potassium iodide aquatic solution is necessary to dissolve iodine), castor oil, and acetone were prescribed most often. These excipients were searched in German and USA professional literature.

The DAC/NRF and USP Compounding Compendium formulations of topical solutions contain the following excipients most frequently prescribed in the analyzed prescriptions of Latvian pharmacies: purified water, ethanol, and castor oil. Acetone, which is used in Latvia for the preparation of Castellani solution, is used also in the USA; in the USP Compounding Compendium, it is included in the composition of carbol–fuchsin topical solution. At present, the DAC/NRF contains several formulations of topical solutions, where glycerol and potassium iodide are used as excipients, but they are not intended for dermatological indications. Latvia does not use several of the solvents often used in formulations in the DAC/NRF, for example, isopropyl alcohol, octyldodecanol, and propylene glycol [10,11].

### 3.5. Topical Powders, Active Ingredients Used in Their Preparation, and Comparison with German and USA Formulations

In the analyzed prescriptions of Latvian pharmacies, nine active ingredients were used in the preparation of topical powders. The most commonly prescribed combination contained two active ingredients from the group of sulfonamides—sulfanilamide and sulfathiazole in equal proportions (85 prescriptions for topical powders or 88.54%). As already mentioned, the DAC/NRF and USP Compounding Compendium do not contain formulations with these active ingredients [10,11].

### 3.6. Oral Solutions, Active Ingredients Used in Their Preparation, and Comparison with German and USA Formulations

In the analyzed prescriptions of Latvian pharmacies, five active ingredients were used in the preparation of oral solutions. Sodium thiosulfate solution (43 prescriptions), calcium chloride (11 prescriptions), and their combinations (27 prescriptions) were prescribed most often. Both active ingredients are prescribed for oral use in the case of allergic diseases. Out of these combinations, most of the prescriptions contained only the said two active ingredients (21 prescriptions); however, a third active ingredient (sodium bromide) was present in six prescriptions. Purified water was used in all the prescriptions as a solvent. Purified water was the only excipient; antimicrobial preservatives were not added to prescribed oral solutions.

The DAC/NRF does not contain oral solutions with sodium thiosulfate as an active ingredient; it contains only one potassium iodide oral drop formulation, where sodium thiosulfate is added as an excipient [10]. The USP Compounding Compendium also does not contain a sodium thiosulfate oral solution [11]. However, this substance is mentioned in USA extemporaneous literature. Sodium thiosulfate is mentioned as an antioxidant for aqueous systems [6]; a preparation of sodium thiosulfate–sodium nitrite solution is also described, which is used against radiological agents [5]. Oral calcium chloride solution, similarly to sodium thiosulfate solution, is neither included in the DAC/NRF nor in the USP Compounding Compendium [10,11].

## 4. Discussion

The results of the survey show that Latvian dermatovenerologists prescribe active ingredients currently used in Germany and the USA, as well as active ingredients, the use of which in dermatology is subject to discussions. For example, boric acid can be used in Germany only as an excipient for preparation of individual non-dermatological dosage forms, because, after topical application of boric acid, excretion is slow and causes the risk of cumulative toxicity, and the effect of boric acid in nontoxic concentrations is controversial [46]. In Latvia, boric acid is widely prescribed in the composition of dermatological dosage forms. The situation is similar also in other European countries, where boric acid can be found in the composition of several topical products. In the composition of individual extemporaneous formulations used in Hungary for dermatological indications, boric acid and borax are prescribed as active ingredients [47]. In the Czech Republic, boric acid preparations are also widely used in dermatology, where solutions and ointments containing boric acid are available on the market [46]. To be noted, Italy has industrially manufactured antiseptic solutions with boric acid, but Poland produces powder, where boric acid is used as one of the active ingredients [48]. Despite the topical adverse reactions after topical application of boric acid, its use is reported [46].

Sulfathiazole can be mentioned as another substance subject to discussion, which is one of the most common active ingredients used in suspensions in Latvia, while, in the USA, sulfathiazole is classified as an unsafe or not effective drug product and is included in the Food and Drug Administration Negative List. Therefore, in the USA, sulfathiazole may not be used for human drug compounding [5]. The situation is similar in Germany, where the sulfathiazole monograph was removed from the DAC/NRF more than a quarter of a century ago [10].

In Latvia, topical dosage forms containing two or more active ingredients were widely prescribed. Up to seven active ingredients have been prescribed in the analyzed prescriptions. Similar data were obtained in Lithuania, where semi-solid dosage forms were prescribed most often, but 48% of all the analyzed prescriptions contained two or more active ingredients [49]. A large number of active ingredients causes the risk that the prescribed ingredients interact among themselves or with any of the excipients [9]. Therefore, standardized formulations mainly contain one to two active ingredients, as we can see from the analysis of the standardized compounded preparation monographs included in the DAC/NRF and USP Compounding Compendium [10,11].

Despite the relatively large diversity of active ingredients in dosage forms prescribed by Latvian dermatovenerologists, the range of excipients is not wide. For example, in suspensions and topical solutions for antimicrobial purposes, only ethanol and glycerol were predominantly used. German and USA professional literature offers a much broader range of antimicrobial preservatives for topical preparations; for instance, other monovalent alcohols such as isopropyl alcohol and benzyl alcohol are offered in addition to ethanol, as well as propylene glycol [6,10]. As described above, oral solutions contained only one vehicle—purified water. For antimicrobial reasons, it is essential to protect prepared oral solutions, whereby antimicrobial preservatives must be added to preparations.

The research revealed that Latvian dermatovenerologists frequently prescribed industrially manufactured finished dosage forms—ointments, creams, solutions, oral capsules, and tablets—in the composition of extemporaneous medicines. The USA and other European countries, for example, the Czech Republic, also have such practices [5,50]. When using a manufactured drug product as a source of active ingredient, it should be taken into account that all commercially available medications also contain excipients, which may affect the efficacy, safety, and stability of the final compounded preparation. For example, if many tablets are prescribed as part of an ointment, then the excipients in the tablets may considerably affect the consistency of the ointment and make the ointment too thick to use conveniently. When adding industrially manufactured ointments to an extemporaneously compounded dosage form, it should be ascertained that phases of the ointments and bases prescribed in the prescription are compatible. It should be taken into account that, when adding industrially manufactured solutions to a prescription, their pH affects the pH of the final compounded preparation [5]. Since industrially manufactured preparations create additional risks, several countries implemented measures to reduce the use of these preparations in extemporaneous compounding. For example, a project was implemented in the Czech Republic, the purpose of which was to provide pharmacies in the Czech Republic with bulk drug substances necessary for the preparation of extemporaneous medicines, which were absent on the national market. The project envisaged the possibility to purchase bulk drug substances in small packages, which is very important for the pharmacies preparing medicines in small amounts [50].

Since compositions prescribed by Latvian dermatovenerologists are often not standardized, prescriptions should be carefully evaluated by pharmacists before preparation to eliminate incompatibility. For example, in the analyzed prescriptions of semi-solid dosage forms, we found erythromycin in combination with lactic acid. According to German professional literature, erythromycin is incompatible with this acid, because stability of erythromycin depends on the pH environment and it is inactivated when the pH is less than 6 [51]. This example illustrates the need to investigate incompatibility problems in a further study.

Not only Latvian dermatovenerologists prescribe extemporaneous medicines, compositions of which are not standardized. Although Germany and the USA have standardized compounded preparation monographs, physicians still prescribe extemporaneous prescriptions for individual patients not included in the compendium or formulary [52,53]. Such formulations require special attention of pharmacists and physicians. German literature describes several examples, when incompatibility of ingredients was identified as a result of cooperation between a pharmacist and a dermatovenerologist, and the prescribed composition was corrected [9,54].

Analyzed prescriptions show deviations of prescription trends from the USA and German norms. This can be partially explained by history of Latvia. In Latvia, as in the former Union of Soviet Socialist Republics (hereinafter referred to as USSR), until the collapse of the USSR, the preparation of extemporaneous medicines was carried out in accordance with uniform procedures and regulatory enactments adopted by the USSR. Almost 30 years passed since Latvia regained independence, but some active ingredients and combinations of active ingredients mentioned in books of that time are still prescribed in Latvia, such as suspensions where sulfathiazole is combined with boric acid and sulfur [55]. Historically used substances could be associated with health risks. Another reason is the limited import of some medicines. For instance, an industrially manufactured ointment with two active ingredients (salicylic acid and mometasone furoate) was not available on the Latvian market for some time, which is why specialists prescribed an extemporaneous composition, which consisted of two ingredients—industrially manufactured mometasone furoate ointment and salicylic acid. Another possible reason is the differences in offered information sources, because Latvia, unlike Germany and the USA, does not have any officially approved standardized compounded preparation monographs.

In order to ensure safe and effective use of extemporaneous medicines for Latvian patients, it is necessary to prescribe those active ingredients and combinations of active ingredients, for which their use in dermatology is evidence-based. Adopting the German and USA experiences would be the first step in the creation of standardized formulations. Since the dermatovenerologist and the pharmacist are jointly responsible for the quality of prescribed and prepared medicines, it is feasible to organize seminars and other further education activities, where pharmacists and dermatovenerologists would be educated on standardized, proven extemporaneous formulations.

## 5. Conclusions

More than half of topical dosage form prescriptions prescribed by dermatovenerologists in Latvia contained two or more active ingredients. The most frequently prescribed number of active ingredients differed depending on the dosage form. Semi-solid dosage forms most commonly contained two active ingredients (32.56%), while suspensions contained four active ingredients (46.34%), topical solutions contained one active ingredient (45.85%), and topical powders contained two active ingredients (94.80%).

The analyzed prescriptions of Latvian dermatovenerologists contained active ingredients used in Germany and the USA, as well as active ingredients, the use of which in Germany and the USA is limited and is permissible only in exceptional cases.

Along with bulk drug substances, industrially manufactured preparations were also used, such as ointments, creams, tablets, capsules, and solutions.

The excipients most commonly used in the analyzed prescriptions of Latvian pharmacies can also be found in the DAC/NRF or USP Compounding Compendium. Extemporaneous dosage forms did not contain many excipients, but their numbers significantly increased in prescriptions, which used industrially manufactured preparations such as tablets, creams, and ointments.

## Figures and Tables

**Table 1:** Number of active ingredients in the semi-solid dosage forms prescribed by dermatovenerologists.

Number of Active Ingredients	Number of Extemporaneous Prescriptions (*n* = 1032)
0 active ingredients	6 (0.58%)
1 active ingredient	224 (21.71%)
2 active ingredients	336 (32.56%)
3 active ingredients	241 (23.35%)
4 active ingredients	128 (12.40%)
5 active ingredients	80 (7.75%)
6 active ingredients	17 (1.65%)
7 active ingredients	0 (0%)

**Table 2:** Number of active ingredients in the suspensions prescribed by dermatovenerologists.

Number of Active Ingredients	Number of Extemporaneous Prescriptions (*n* = 820)
0 active ingredients	0 (0%)
1 active ingredient	12 (1.46%)
2 active ingredients	118 (14.39%)
3 active ingredients	204 (24.88%)
4 active ingredients	380 (46.34%)
5 active ingredients	83 (10.12%)
6 active ingredients	16 (1.95%)
7 active ingredients	7 (0.86%)

**Table 3:** Number of active ingredients in the topical solutions prescribed by dermatovenerologists.

Number of Active Ingredients	Number of Extemporaneous Prescriptions (*n* = 482)
0 active ingredients	14 (2.91%)
1 active ingredient	221 (45.85%)
2 active ingredients	130 (26.97%)
3 active ingredients	98 (20.33%)
4 active ingredients	19 (3.94%)
5 active ingredients	0 (0%)
6 active ingredients	0 (0%)
7 active ingredients	0 (0%)

**Table 4:** Number of active ingredients in the topical powders prescribed by dermatovenerologists.

Number of Active Ingredients	Number of Extemporaneous Prescriptions (*n* = 96)
0 active ingredients	0 (0%)
1 active ingredient	2 (2.08%)
2 active ingredients	91 (94.80%)
3 active ingredients	1 (1.04%)
4 active ingredients	2 (2.08%)
5 active ingredients	0 (0%)
6 active ingredients	0 (0%)
7 active ingredients	0 (0%)

**Table 5:** Number of active ingredients in the oral solutions prescribed by dermatovenerologists.

Number of Active Ingredients	Number of Extemporaneous Prescriptions (*n* = 89)
0 active ingredients	0 (0%)
1 active ingredient	56 (62.92%)
2 active ingredients	27 (30.34%)
3 active ingredients	6 (6.74%)
4 active ingredients	0 (0%)
5 active ingredients	0 (0%)
6 active ingredients	0 (0%)
7 active ingredients	0 (0%)

**Table 6:** Active ingredients used in preparation of semi-solid dosage forms.

No.	Active Ingredient	Number of Extemporaneous Prescriptions (*n* = 1032)
1	Salicylic acid	621 (60.17%)
2	Sulfur for external use	448 (43.41%)
3	Prednisolone (tablets)	159 (15.41%)
4	Zinc oxide	147 (14.24%)
5	Metronidazole (tablets)	140 (13.57%)
	Metronidazole (bulk drug substance)	1 (0.1%)
6	Dexamethasone (tablets)	74 (7.17%)
	Dexamethasone sodium phosphate (solution for injection)	1 (0.1%)
7	Birch tar	73 (7.07%)
8	Boric acid	65 (6.30%)
9	Yellow mercuric oxide	60 (5.81%)
10	Ampicillin trihydrate (capsules)	55 (5.33%)
11	Diphenhydramine hydrochloride	52 (5.04%)
12	Ichthammol	52 (5.04%)
13	Benzocaine	47 (4.55%)
14	Bismuth subgallate	42 (4.07%)
15	Resorcinol	35 (3.39%)
16	Lactic acid	34 (3.29%)
17	Turpentine oil, *Pinus pinaster* type	30 (2.91%)
18	Procaine hydrochloride	27 (2.62%)
19	Erythromycin (tablets)	21 (2.03%)
20	Menthol	18 (1.74%)
21	Chloramphenicol	10 (0.97%)

**Table 7:** Classification of industrially manufactured ointments and creams used in preparation of semi-solid dosage forms by pharmacotherapeutic group.

ATC Code, Active Ingredient(s), and Dosage Form of the Industrially Manufactured Preparation	Number of Extemporaneous Prescriptions (*n* = 1032)
**Imidazole and Triazole Derivatives (D01AC)**	
Clotrimazole cream	43 (4.17%)
Isoconazole nitrate cream	2 (0.19%)
Fenticonazole nitrate cream	1 (0.1%)
**Imidazoles/Triazoles in Combination with Corticosteroids (D01AC20)**	
Isoconazole nitrate and diflucortolone valerate cream	79 (7.66%)
**Emollients and Protectives: Carbamide Products (D02AE)**	
Urea cream	1 (0.1%)
**Preparations for Treatment of Wounds and Ulcers (D03)**	
Dexpanthenol cream	14 (1.36%)
Dexpanthenol ointment	1 (0.1%)
Dialysate from calf blood ointment	1 (0.1%)
**Corticosteroids, Plain (D07A)**	
Fluocinolone acetonide ointment	38 (3.68%)
Betamethasone valerate ointment	30 (2.91%)
Mometasone furoate cream	29 (2.81%)
Mometasone furoate ointment	7 (0.68%)
Clobetasol propionate cream	3 (0.29%)
Fluticasone propionate cream	3 (0.29%)
Hydrocortisone acetate ointment	3 (0.29%)
Clobetasol propionate ointment	1 (0.1%)
Fluticasone propionate ointment	1 (0.1%)
Triamcinolone acetonide ointment	1 (0.1%)
**Corticosteroids, Combinations with Antiseptics (D07B)**	
Flumetasone pivalate and Clioquinol ointment	1 (0.1%)
**Corticosteroids, Combinations with Antibiotics (D07C)**	
Fusidic acid and Betamethasone valerate cream	14 (1.36%)
**Anti-Acne Preparations for Topical Use (D10A)**	
Adapalene cream	1 (0.1%)
**Other Dermatological Preparations (D11A)**	
Hydroquinone ointment	1 (0.1%)

ATC—Anatomical Therapeutic Chemical

**Table 8:** Excipients used in preparation of semi-solid dosage forms. DAC—Deutscher Arzneimittel-Codex.

No.	Excipient	Number of Extemporaneous Prescriptions (*n* = 1032)
1	Soft paraffin	709 (68.7%)
2	Wool fat	517 (50.1%)
3	Purified water	250 (24.22%)
4	Sunflower oil	220 (21.32%)
5	Potato starch	105 (10.17%)
6	Wolff Basis Creme or Basiscreme DAC	81 (7.85%)
7	Olive oil	69 (6.69%)
8	Liquid paraffin	39 (3.78%)
9	Castor oil	15 (1.45%)
10	Glycerol	5 (0.48%)
11	Peach oil	5 (0.48%)
12	Essex hydrogel	4 (0.39%)
13	Ethanol	3 (0.29%)
14	Talc	3 (0.29%)

**Table 9:** Active ingredients used in preparation of suspensions.

No.	Active Ingredient	Number of Extemporaneous Prescriptions (*n* = 820)
1	Boric acid	674 (82.2%)
2	Salicylic acid	615 (75.0%)
3	Sulfur for external use	523 (63.78%)
4	Camphor	391 (47.68%)
5	Sulfathiazole	121 (14.76%)
6	Zinc oxide	104 (12.68%)
7	Chloramphenicol	80 (9.76%)
8	Resorcinol	67 (8.17%)
9	Menthol	55 (6.71%)
10	Sulfanilamide	54 (6.59%)
11	Diphenhydramine hydrochloride	48 (5.85%)
12	Acetic acid	33 (4.02%)
13	Calendula tincture	33 (4.02%)
14	Metronidazole (tablets)	22 (2.68%)
	Metronidazole (bulk drug substance)	1 (0.12%)
15	Lactic acid	19 (2.32%)
16	Benzocaine	16 (1.95%)
17	Iodine tincture 5%	14 (1.71%)
18	Dimethyl sulfoxide	13 (1.59%)
19	Terbinafine hydrochloride (tablets)	13 (1.59%)

**Table 10:** Excipients used in preparation of suspensions.

No.	Excipient	Number of Extemporaneous Prescriptions (*n* = 820)
1	Purified water	763 (93.05%)
2	Ethanol	752 (91.71%)
3	Glycerol	604 (73.66%)
4	Talc	98 (11.95%)
5	Ether	25 (3.05%)
6	Potato starch	9 (1.1%)
7	Sunflower oil	7 (0.85%)
8	Castor oil	6 (0.73%)
9	Olive oil	5 (0.61%)
10	Peppermint water	2 (0.24%)
11	Citral	1 (0.12%)
12	Lavender oil	1 (0.12%)

**Table 11:** Active ingredients used in preparation of topical solutions.

No.	Active Ingredient	Number of Extemporaneous Prescriptions (*n* = 482)
1	Acetic acid	202 (41.91%)
2	Boric acid	134 (27.8%)
3	Salicylic acid	126 (26.14%)
4	Iodine	116 (24.07%)
	Iodine tincture 5%	4 (0.83%)
5	Resorcinol	90 (18.67%)
6	Benzoic acid	51 (10.58%)
7	Phenol, liquefied	29 (6.02%)
8	Chloramphenicol	16 (6.02%)
9	Fuchsin	15 (3.11%)
10	Borax	12 (2.49%)

**Table 12:** Excipients used in preparation of topical solutions.

No.	Excipient	Number of Extemporaneous Prescriptions (*n* = 482)
1	Purified water	382 (79.3%)
2	Ethanol	323 (67.01%)
3	Glycerol	278 (57.68%)
4	Potassium iodide	35 (7.26%)
5	Castor oil	31 (6.43%)
6	Acetone	29 (6.02%)
7	Hydrochloric acid	28 (5.81%)
8	Sunflower oil	12 (2.49%)
9	Citral	4 (0.83%)
10	Peppermint water	3 (0.62%)
11	Ether	1 (0.21%)
